# Manual Registration in AR-Assisted Surgical Navigation: A Comparative Evaluation

**DOI:** 10.1007/s11548-025-03410-4

**Published:** 2025-06-25

**Authors:** Jiaqi Tang, Abdullah Thabit, Theo van Walsum, Ricardo Marroquim, Mohamed Benmahdjoub

**Affiliations:** 1 Department of Radiology & Nuclear Medicine, Erasmus MC, University Medical Center Rotterdam, Rotterdam, The Netherlands; 2 Computer and Graphics Visualization Group, Delft University of Technology, Delft, The Netherlands

**Keywords:** Augmented Reality, Surgical Navigation, Manual Registration, Hybrid Tracking

## Abstract

**Purpose:** This study evaluates two virtual auxiliary tools, degrees of freedom (DOF) Separation and PinNPivot, to address depth perception limitations and high error rates in manual registration for AR-assisted surgical navigation.

**Methods:** DOF Separation decouples translation and rotation using six independent controls, minimizing cumulative errors. PinNPivot constrains object motion around virtual pins to stabilize rotation. Their effectiveness in AR remains underexplored. Using a hybrid evaluation system (Vuforia and NDI optical tracking), these tools were compared to unassisted manual registration on two patient-specific phantoms, assessing accuracy, task completion time, and NASA-TLX workload scores.

**Results:** PinNPivot balanced efficiency and accuracy but was prone to initial pin placement errors. DOF Separation achieved the highest accuracy but required longer task times due to iterative adjustments. NASA-TLX results showed higher cognitive and physical workload for assisted methods.

**Conclusion:** DOF Separation and PinNPivot improved registration accuracy and efficiency over unassisted manual registration. As software-based tools requiring no additional hardware, they hold promise for enhancing AR-assisted surgical navigation. Future work should validate their clinical applicability in diverse scenarios.

## Introduction

Augmented reality (AR) is increasingly adopted in surgical navigation to provide real-time visualization and spatial alignment of virtual anatomical models with patient-specific anatomy. Accurate registration between the virtual and real environments is critical for ensuring precise guidance during surgery [1]. While automatic registration techniques have made significant progress, they often require fiducial markers and suffer from reduced accuracy due to tissue deformation and motion artifacts [2]. These limitations necessitate manual registration approaches, allowing surgeons to perform real-time interactive adjustments. However, manual registration in AR remains challenging due to inherent depth perception limitations, interaction instability, and the absence of tactile feedback, leading to increased positional and rotational errors [3, 4].

To mitigate these challenges, auxiliary interaction tools such as PinNPivot and degrees of freedom (DOF) Separation have been introduced to improve the precision and usability of manual registration methods. PinNPivot enables users to anchor one or two virtual pins onto an object, constraining movement to a defined pivot point or an axis formed by two pins. This approach stabilizes rotational alignment and enhances depth perception by reducing degrees of freedom in manual adjustments [5]. DOF Separation, on the other hand, decouples translation and rotation into independent axes, allowing users to make fine-grained adjustments along specific degrees of freedom, thereby minimizing unintended misalignment during registration tasks [6]. While these tools have been validated in virtual reality (VR) for improving object manipulation, their effectiveness in AR-assisted surgical navigation remains underexplored.

Previous research has primarily focused on assessing registration accuracy, but practical implementation in clinical settings requires a broader evaluation, incorporating efficiency and usability considerations [7, 8]. This study systematically investigates the impact of PinNPivot and DOF Separation on AR-assisted manual registration by comparing them against unassisted registration. Using a hybrid tracking system integrating Vuforia and NDI optical tracking, we evaluate these tools on patient-specific phantoms, analyzing their impact on positional and rotational errors, task completion time, and user experience. Our findings aim to provide insights into optimizing manual registration workflows and facilitating the adoption of AR-based navigation in clinical applications.

## Methods

Manual registration accuracy in AR-assisted surgical navigation was evaluated using two patient-specific phantoms and a hybrid system combining an NDI Polaris Vega optical tracker (Northern Digital Inc., Canada) and HoloLens 2 (Microsoft Corp., USA) with Vuforia engine (version 10.2). Three methods were tested, unassisted manual registration, DOF Separation, and PinNPivot, assessing positional and rotational errors, task completion times, and user feedback.

**Fig. 1:**
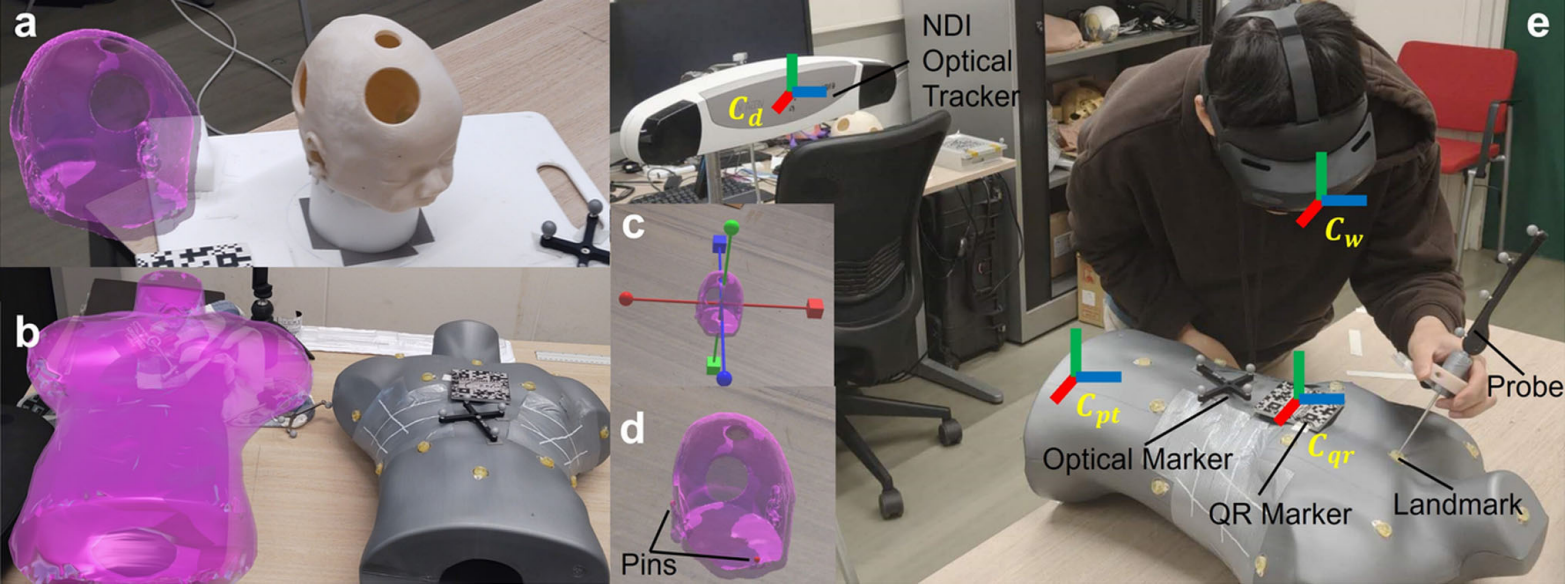
Experimental setup: (a) infant head; (b) adult torso. (c, d) Widgets used for DOF Separation and PinNPivot. (e) The evaluation system integrates Vuforia and NDI Optical Tracker

Two phantoms with CT scans available were used: an infant head model with 10 divots and an adult torso model with 12 conical fiducial markers (PinPoint 128, Beekley). These landmarks, predefined in the patient coordinate system ($C_{pt}$) from CT space, served as reference points for evaluating registration accuracy.

As shown in Fig. 1, each phantom featured a QR marker and an optical marker for tracking. The Vuforia engine tracked the QR marker’s pose in the HoloLens 2 world coordinate system ($C_{w}$), while the NDI tracker recorded the optical marker’s pose in its device coordinate system ($C_{d}$). A fixed transformation ($T_{d-w}$) enabled the conversion of optical marker coordinates from $C_{d}$ into $C_{w}$. A probe equipped with optical markers was used to localize physical landmarks, recording their coordinates in $C_{d}$. These were transformed into $C_{qr}$, the QR marker’s coordinate system, to define the ground truth ($P_{qr}$). Virtual phantom landmarks ($\tilde{P}_{qr}$), sharing geometry with the physical phantom, were similarly transformed for comparison.

To quantitatively evaluate registration accuracy, positional and rotational errors were computed. The centroids of the physical and virtual landmark sets, $P_{qr}$ and $\tilde{P}_{qr}$, were first determined:

1 $$\begin{aligned} c_{qr} = \frac{1}{N} \sum _{i=1}^{N} P_{qr}^{(i)}, \quad \tilde{c}_{qr} = \frac{1}{N} \sum _{i=1}^{N} \tilde{P}_{qr}^{(i)} \end{aligned}$$

where $c_{qr}$ and $\tilde{c}_{qr}$ represent the centroids of the physical and virtual landmark sets, respectively. The translational error *t* was then calculated as:

2 $$\begin{aligned} t = \Vert \tilde{c}_{qr} - c_{qr} \Vert \end{aligned}$$

To further quantify rotational accuracy, we computed the optimal rotation matrix *R* aligning the virtual landmarks to the physical landmarks:

3 $$\begin{aligned} P_{qr} - c_{qr} = R (\tilde{P}_{qr} - \tilde{c}_{qr}) \end{aligned}$$

where *R* is the best-fit rotation matrix obtained through singular value decomposition (SVD). The rotational error $\theta$ was then determined using:

4 $$\begin{aligned} \theta = \arccos \left( \frac{\text {Trace}(R) - 1}{2}\right) \end{aligned}$$

where $\text {Trace}(R) = R_{11} + R_{22} + R_{33}$ is the sum of the diagonal elements of the rotation matrix.

Nine participants with prior experience in VR/AR interaction performed registration tasks on both phantoms using the three methods. Before the formal trials, participants completed a training session to familiarize themselves with each registration method. To ensure a fair evaluation and reduce learning effects, a counterbalanced experimental design was implemented, where each participant performed all three registration tasks in a randomized order. This design minimizes potential biases arising from task sequence, ensuring that observed differences in performance are attributable to the registration methods rather than familiarity or fatigue. Each trial began with eye calibration to enhance depth perception consistency, and the virtual model was initialized with a fixed offset in position and rotation. The order of phantoms and registration methods was randomized, and task completion times, as well as positional and rotational registration errors, were recorded.

## Results

**Fig. 2:**
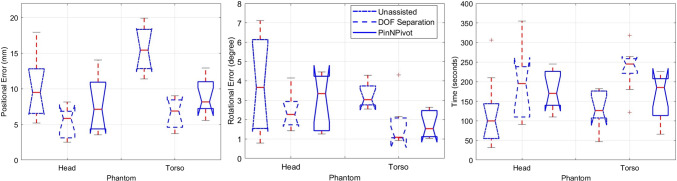
Boxplots of positional errors (left), rotational errors (middle), and task completion times (right) for the three methods on head and torso phantoms. Results are based on 9 participants, showing the performance difference across the methods and phantoms

The performance of the three registration methods was evaluated based on positional errors, rotational errors, and task completion times across the two phantoms. As shown in Fig. 2, unassisted manual registration resulted in the shortest task times but had the highest positional and rotational errors, particularly on the larger torso phantom, where depth perception and alignment challenges were more pronounced.

**Fig. 3:**
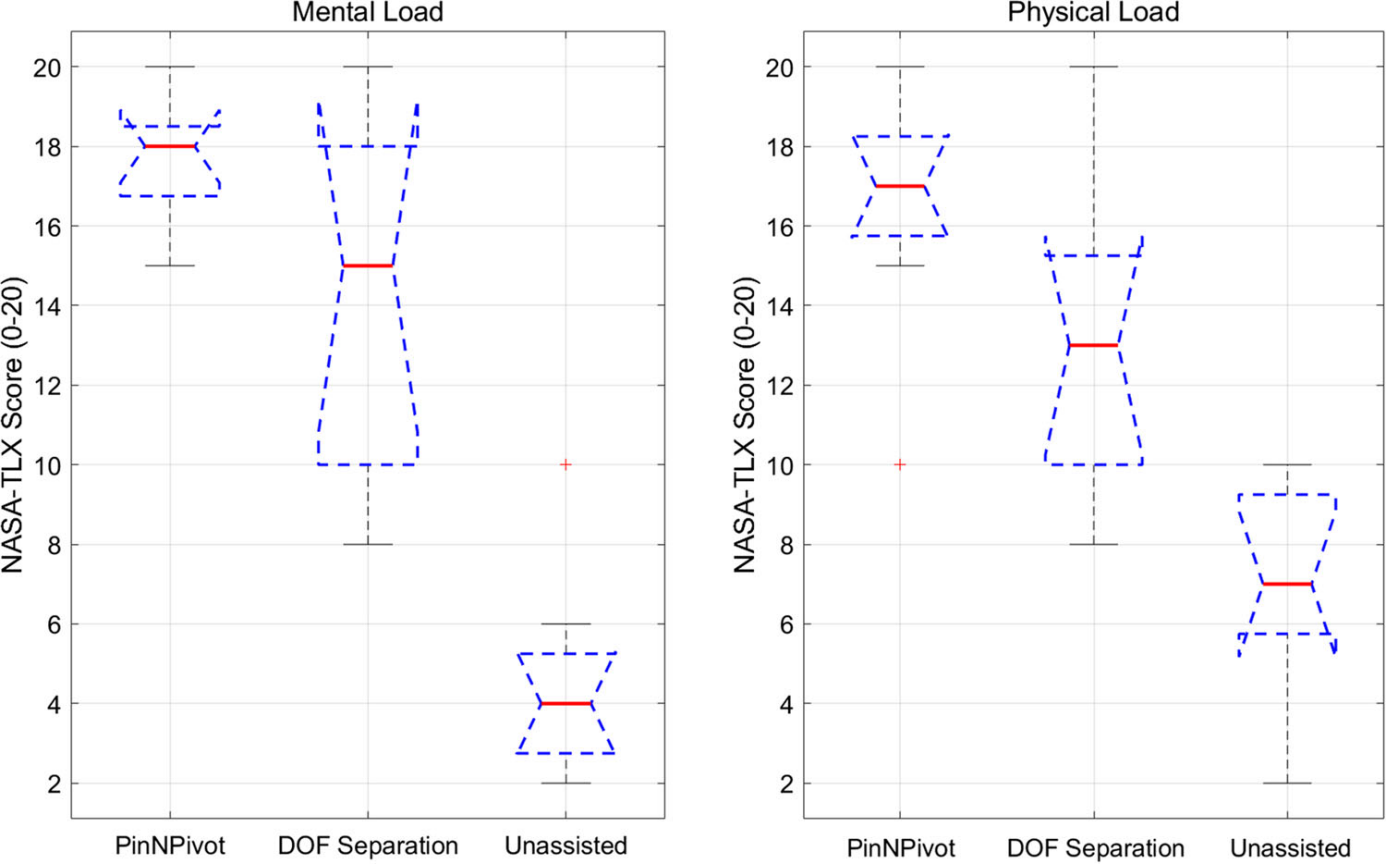
NASA-TLX scores for mental(left) and physical(right) workload across the three registration methods. Boxplots show the distribution of responses among the nine participants

DOF Separation achieved the highest accuracy, with positional errors reduced by 45% and rotational errors by 38% compared to unassisted manual registration. However, it required the longest task times due to iterative adjustments. The fine-grained control over translation and rotation allowed for precise alignment but increased cognitive and motor workload. The performance difference between DOF Separation and PinNPivot was more pronounced on the head phantom, where smaller anatomical structures required more precise manipulation. On the torso phantom, DOF Separation still had the lowest errors, but the performance gap between it and PinNPivot was smaller, suggesting that increased interaction complexity may limit usability in larger anatomical regions.

PinNPivot provided a balance between accuracy and efficiency, with positional and rotational errors reduced by 30% and 25%, respectively, while maintaining shorter task times than DOF Separation. By constraining movement along selected axes, it simplified the alignment process while still offering improved accuracy over unassisted manual registration. The boxplots show that PinNPivot’s accuracy was closer to DOF Separation on the torso phantom, likely because the larger structure benefitted from its constrained interactions. On the head phantom, where higher precision was needed, DOF Separation had a clearer advantage.

Figure 3 shows the NASA-TLX workload ratings, highlighting differences in mental and physical demands across methods. PinNPivot had the highest mental workload due to its constrained movement, followed by DOF Separation, while unassisted registration was the least mentally demanding. In terms of physical effort, DOF Separation was the most demanding due to its iterative adjustments, with PinNPivot slightly lower and unassisted registration requiring the least effort. These results reflect a trade-off between accuracy and user effort.

## Discussion

DOF Separation and PinNPivot significantly improve manual registration accuracy in AR-assisted surgical navigation compared to unassisted methods. DOF Separation achieved the highest accuracy but required longer task times due to its iterative adjustments, increasing cognitive and motor workload. In contrast, PinNPivot provided a balance between accuracy and efficiency by constraining motion, allowing faster adjustments while maintaining substantial accuracy improvements. However, initial pin placement errors could propagate, making corrections difficult.

While this study demonstrated the potential of these auxiliary tools, a key limitation is the relatively small sample size (N=9), which may affect the statistical power of the results. Although the experimental design aimed to minimize biases through counterbalancing, a larger participant pool would provide a more comprehensive evaluation of inter-user variability and improve the generalizability of the findings. Future studies should involve a more diverse cohort, including clinicians with varying levels of experience, to assess the broader applicability of these methods in clinical settings.

A hybrid approach combining PinNPivot for initial alignment with DOF Separation for fine-tuning could leverage their respective strengths, improving both efficiency and precision. Participant feedback highlighted usability challenges, including occasional hand-tracking failures in PinNPivot and unintended transformations in DOF Separation due to fixed handle orientations. Addressing these issues through improved tracking robustness and adaptive interface design could enhance usability.

Although this study was conducted on rigid phantoms, the experimental setup was designed to closely resemble real-world surgical workflows. Manual registration remains crucial when intraoperative tissue deformation requires real-time adjustments. The use of auxiliary tools such as PinNPivot and DOF Separation has the potential to improve spatial alignment and mitigate depth perception challenges in AR-assisted navigation. Future work should explore how these methods perform with deformable tissue models, potentially integrating real-time intraoperative adaptation techniques.

## Conclusion

This study demonstrates the potential of PinNPivot and DOF Separation to enhance manual registration accuracy in AR-assisted surgical navigation. DOF Separation exhibited the highest accuracy, effectively reducing positional and rotational errors, but at the cost of increased cognitive and physical workload. PinNPivot, while slightly less precise, offered a more balanced trade-off between accuracy and efficiency, reducing task completion time and maintaining reasonable precision.

NASA-TLX assessments revealed that both assisted methods increased cognitive and physical workload compared to unassisted manual registration. These findings emphasize the need to further refine interaction techniques to improve usability while maintaining accuracy. As purely software-based solutions requiring no additional hardware, these tools show promise for integration into clinical AR workflows.

Future work should focus on validating their effectiveness in real-world surgical scenarios, particularly in dynamic environments with deformable tissues. Additionally, further improvements in tracking robustness, user interface design, and adaptive interaction strategies could help mitigate workload while maximizing accuracy.
